# Salinity tolerance in resting cysts of colpodid ciliates: Comparative transcriptomics analysis and chemical analysis of cyst walls to investigate their tolerance capability

**DOI:** 10.1016/j.crmicr.2025.100371

**Published:** 2025-03-07

**Authors:** Ryota Saito, Hiroki Yamanobe, Kazuma Yabuki, Tomohiro Suzuki, Takeru Saito, Shuntaro Hakozaki, Manfred Wanner, Ryota Koizumi, Tatsuya Sakai, Maribet Gamboa, Toshihiko Tanaka, Akiko Ono, Hoa Thanh Nguyen, Yuta Saito, Tetsuya Aoyama, Katsuhiko Kojima, Futoshi Suizu, Kozo Watanabe, Yoichiro Sogame

**Affiliations:** a Department of Applied Chemistry and Biochemistry, National Institute of Technology, Fukushima College, Iwaki, Fukushima 970-8034, Japan; b Present address: Department of Chemistry and Biotechnology, Kochi University, Kochi, Kochi 780-8520, Japan; c Present address: College of Biological Sciences, University of Tsukuba, Tsukuba, Ibaraki 305-8577, Japan; d Center for Bioscience Research and Education, Utsunomiya University, Utsunomiya, Utsunomiya 321-8505, Japan; e Present address: College of Agri-Biological Resource Sciences, University of Tsukuba, Tsukuba, Ibaraki 305-8577, Japan; f Present address: Faculty of Symbiotic Systems Science, Fukushima University, Fukushima, Fukushima 960-1296, Japan; g Brandenburg University of Technology Cottbus-Senftenberg, Department of Ecology, Cottbus D-03013, Germany; h Department of Ecology, Faculty of Science, Universidad Catolica de la Santisima Concepcion, Alonso de Ribera 2850, Concepcion, Chile; i Centro de Investigación en Biodiversidad y Ambientes Sustentables (CIBAS), Universidad Católica de la Santísima Concepción, Alonso de Ribera 2850, Concepción, Chile; j Hamamatsu University School of Medicine, Hamamatsu, Shizuoka 431-3192, Japan; k Elements Chemistry Laboratory, RIKEN Cluster for Pioneering Research (CPR), Wako, Saitama 351-0198, Japan; l Ultrahigh Precision Optics Technology Team, RIKEN Center for Advanced Photonics (RAP), Wako, Saitama 351-0198, Japan; m Present address: Faculty of Global Interdisciplinary Science and Innovation, Shizuoka University, Shizuoka, Shizuoka 422-8529, Japan; n Center for Marine Environmental Studies (CMES), Ehime University, Matsuyama, Ehime 790-8577, Japan; o Present address: Department of Biomedical Sciences, School of Medicine and Health Sciences, University of North Dakota, Grand Forks, North Dakota, USA; p Department of Microbiology and Immunology, Shinshu University School of Medicine, Matsumoto, Nagano 390-8621, Japan; q Molecular Oncologic Pathology, Department of Pathology and Host-Defense, Faculty of Medicine, Kagawa University, Takamatsu, Kagawa 761-0793, Japan; r Present address: Laboratory of Pathology, Department of Medical Technology, Kagawa Prefectural University of Health Sciences, Takamatsu, Kagawa 761-0123, Japan

**Keywords:** Protist, Chitin, Actin, NGS, Dormancy, Cryptobiosis, Biodiversity

## Abstract

The formation of resting cysts is a strategy for survival in unfavorable environments by single cell organisms such as protists. Here, we show that *Colpoda* resting cysts have high salinity tolerance and investigate the changes in gene expression that underpin this effect. *Colpoda* resting cysts can tolerate saline conditions up to 3.5 % NaCl. A comparative transcriptome analysis of vegetative cells and resting cysts showed that the relative levels of expression of genes associated with membrane function increased in resting cysts. These changes in gene expression suggest that reconstruction of the plasma membrane is associated with salinity tolerance. The resting cyst forms cyst-specific cellular structure known as the cyst wall. The outer shell-like layer, called the ectocyst, while the inner multiple layers, known as the endocyst. The chemical analysis showed ectocyst contains chitin and endocyst contains several proteins. These structures can protect cells by acting as a biological armor or protective materials. The results of this study offer a possible scenario in which salinity tolerance enables the widespread dispersal of protists.

## Highlights

• Resting cyst formation is a strategy for survival in unfavorable environments.
• *Colpoda* resting cysts can tolerate saline conditions up to 3.5 % NaCl.
• Genes expression associated with membrane function is increased in resting cysts.
• Reconstructing membrane is suggested to contribute to the tolerance.
• Cyst wall: ectocyst contains chitin and endocyst contains actin.

## Graphical abstract

**Figure:**
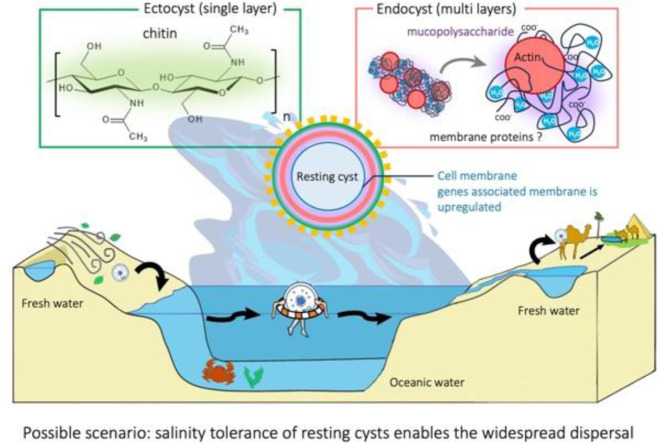

## Introduction

Colpodid ciliates are among the most ubiquitous and diverse organisms in soil (Finlay et al., 1999; Finlay et al., 2001; Fenchel, 2003); hence, they play key functional roles in all ecosystems (Singer et al., 2021). All colpodid ciliates can form resting cysts (Lynn, 2008), a strategy used by protists to survive when their environmental conditions become unfavorable (Gutiérrez et al., 1990, 2001, 2003; Venrni and Rosati, 2011). The ability to form resting cysts may be one of the reasons that colpodid ciliates have survived the drastic environmental changes that have occurred since eukaryotic life emerged (Keeling et al., 2005).

Resting cyst formation entails reversible morphogenetic and physiological changes, which are regulated by differential gene/protein expression (Grisvard et al., 2008; Chen et al., 2014; Gao et al., 2015, 2018; Pan et al., 2019, 2021; Sogame et al., 2012a, 2014a, 2020) and post-transcriptional modification of proteins e.g., protein phosphorylation (Sogame et al., 2011a, 2011b 2012b, 2014b; Sogame and Matsuoka, 2012). The most notable morphogenetic changes are the absorption of thier cilia are absorbed (Funatani et al., 2010; Matsuoka et al., 2021; Chen et al., 2014), disappearance of the mitochondrial membrane potential disappears (Funatani et al., 2010; Sogame et al., 2014a; Hakozaki et al., 2023), the size reduction of both the cell and the macronucleus (Zou et al., 2021), and the disruption of the cytoskeletal structure is disrupted (Sogame et al., 2020; Gao et al., 2015; Chen et al., 2018; Grisvard et al., 2008; Makioka et al., 2012). Physiological changes include increased activity of the ubiquitin proteasome system (Pan et al., 2019: Gallego et al., 2007; Gao et al., 2015; Pan et al., 2021) and of lysosomes (Chen et al., 2014), and the activation of the autophagy system (Jiang et al., 2019; Pan et al., 2019). In contrast to the deconstruction of some morphological structures, cyst formation produces a new cell wall-like structure. In *Colpoda*, the cell is surrounded by a shell-like outer layer (ectocyst) with several inner layers (endocyst) (Goodey, 1913; Kida and Matsuoka, 2006; Funatani et al., 2010). The chemical composition of the cyst wall is largely unknown.

Resting cysts have been shown to be able to tolerate desiccation (Corliss et al., 1974; Goodey, 1915), high and low temperatures (Taylor, 1936), freezing (Uspenskaya et al., 1979; Matsuoka et al., 2020), UV irradiation (Matsuoka et al., 2017), acid and alkali treatments (Sogame et al., 2011c; Nakamura et al., 2020), electrostatic exposure (Saito et al., 2023), and gamma irradiation (Saito et al., 2020a, 2020b). However, their tolerance to salinity has not yet been elucidated. The expectation is that the cysts are tolerant of salinity changes because the protists often inhabit temporary aqueous environments such as soil of puddles (Lynn et al., 2008) that are subject to evaporation and therefore to changes in salinity concentration.

In this study, we examined the salinity tolerance of resting cysts and performed a comparative gene expression analysis between vegetative cells and resting cysts to identify factors involved in tolerance. We also performed a chemical analysis of the cyst wall in order to determine its contribution to salinity tolerance. This study provides novel information on the tolerance of resting cysts and new insights into the significance of resting cyst formation for the ubiquitous distribution and adaptation of protists to a wide range of environments.

## Materials and methods

This study shows tolerance of *Colpoda* resting cysts to high salinity, differential gene expression, and materials of cyst wall by bioassay, microscopy, RNA sequence, attenuated total reflectance infrared spectroscopy (ATR-IR), and Mass spectral analysis (LC-MS/MS).

### Culture, induction of encystment and excystment, sample preparation

*Colpoda cucullus* strain R2TTYS was used for all experiments. Cell culture, cell preparation before experiments, and encystment and excystment induction were basically carried out according to the previous study (Saito et al., 2023) at room temperature at 25 °C. Cells were maintained in a culture medium (infusion of 0.05 % (w/v) dried rice leaves supplemented with 0.05 % Na_2_HPO_4_). After 1 day of culture, cells were collected by centrifugation (1500 x *g* for 1 min), washed twice in 1 mM Tris–HCl (pH 7.2), and used for experiments. Encystment was induced by suspending *C. cucullus* cells at high cell density (10,000–50,000 cells mL^-1^) in encystment-inducing medium [1 mM Tris–HCl (pH 7.2), 0.1 mM CaCl_2_; En-medium]. For RNA extraction, 50 μg/mL (final conc.) ampicillin sodium was added to the samples. Excystment was induced by replacing En-medium with excystment-inducing medium (0.2 % infusion of dried rice leaves supplemented with 0.05 % (w/v) Na_2_HPO_4_; Ex-medium).

### Microscopy

Vegetative cells and cysts of *C. cucullus* were analyzed under an optical microscope Axiovert A1 system (Carl Zeiss Co. Ltd., Tokyo, Japan) or Axioscope A1 system (Carl Zeiss Co. Ltd.). Endocyst staining was performed using toluidine blue (TB) as previously described (Sogame et al., 2011c).

The effects of salinity on the cysts were assessed using a “shrink ratio” as described previously (Nakamura et al., 2020). In brief, micrographs were taken of 1-week-old cysts (Fig. 2A‘C’), those treated with En-medium containing 3.5 % NaCl for a week (Fig. 2A‘T’), and those washed and re-suspended with En-medium after the treatment (Fig. 2A‘R’). The area surrounded by the plasma membrane (PM) and ectocyst (Ec) was measured using ZEN 3.3 blue edition software (Carl Zeiss Co. Ltd.) and an area ratio [area surrounded by PM (μm^2^) / area surrounded by Ec (μm^2^)] was calculated.

For lectin staining, cysts over 2 weeks old were induced to excyst for 6 h. After excystment, the vacant cysts (i.e., ectocysts) were collected, washed three times with 1 mM Tris–HCl and stained with an equal volume of 1 mg/mL wheat germ agglutinin (WGA): FITC conjugated (J-Oil Mills, Inc. Tokyo, Japan) at 4 °C for 30 min. The lectin-stained ectocysts were washed three times with 1 mM Tris–HCl and fluorescent staining was analyzed using an Axioscope A1 system with a 475 nm LED laser.

### Attenuated total reflectance infrared spectroscopy (ATR-IR) analysis

Two-weeks-old cysts were induced to excyst for 6 h and the ectocysts were collected, washed 3 times in distilled water and air-dried. The ATR-IR spectra of the ectocysts were recorded on a JOEL FTIR 6800 spectrometer equipped with a JOEL Pro One Diamond ATR unit. Each ectocyst samples was placed on the diamond prism and the spectra were recorded under a nitrogen atmosphere before and after washing with ethanol for 30 min. Powdered chitin (chitin from shrimp shells, Sigma-Aldrich Janan LLC., Tokyo, Japan) was also measured on the prism to obtain a spectrum for chitin. A reference spectrum was taken with the prism without any samples and was subtracted from each sample spectrum during data processing.

### Bioassays

The cell proliferation assay was basically carried out in a previous study (Saito et al., 2020c). Vegetative cells (including excysted cells) were suspended at a low cell density (500 cells mL^-1^) in 4 mL of fresh Ex-medium (highly concentrated culture medium suppressing cyst formation) containing different concentrations of NaCl (0, 0.3, 1.0, 3.5, 5.0, 10.0, 30.0 % w/v). The NaCl concentration of 0.3 %, 3.5 % and 30.0 % are approximately equivalent to the salinity concentration of brackish water, sea water, and Dead Sea water, respectively (Buchalo et al., 1998; Takagi et al., 2006; Elma et al., 2015). Cell numbers were counted at intervals after initiation of the culture; three aliquots of 20 μL were removed from the samples, cell numbers were counted under a Stemi 305 microscope (Carl Zeiss Co., Ltd., Tokyo, Japan), and the density of cells was calculated.

Cell proliferative capability of excysted cells from cysts exposed to high salinity was evaluated by suspending the cells at a high density in En-medium and incubating them for 1 week. The suspension was then replaced with either En-medium containing 3.5 % NaCl or En-medium. After incubation for a week, the suspensions were replaced with Ex-medium and the cells were incubated for 6 h. Excysted cells were re-suspended in fresh Ex-medium at a low cell density (500 cells mL^-1^). Cell numbers were counted at hourly intervals in a 20 μL aliquot using a Stemi 305 microscope.

To evaluate the tolerance of cysts to salinity, cysts that were >1 week old were prepared in a Petri dish. The medium was replaced with En-medium containing different concentrations of NaCl (0, 0.3, 1.0, 3.5, 5.0, 10.0, 30.0 % w/v), and the cyst samples were incubated for a week. The exposed cysts were washed and re-suspended in a fresh Ex-medium for >6 h and an excystment assay was performed as previously described (Saito et al., 2020c). To evaluate the effect of salinity on excystation, the rate of excystment of 1-week-old cysts, which were induced by Ex-medium with/without NaCl, was calculated.

The effect of saline exposure on encystation was measured as encystment (%) of exposed vegetative cells. The vegetative cells were induced by En-medium with or without 0.3 % NaCl. The encystment rate was calculated as described previously (Saito et al., 2020c).

Statistical analyses were performed using Tukey's test in the Bell Curve for the Excel software package (Social Survey Research Information Co., Ltd., Japan).

### Total RNA extraction, library preparation, and de novo sequencing

Total RNAs were extracted from *C. cucullus* vegetative cells (300,000 cells) and 2-week-old mature cysts according to Kida and Matsuoka (2006) (3000,000 cells) using the Direct-zol RNA purification system (Zymo Research Crop., California, USA) according to the manufacturer's instructions (Hakozaki et al., 2023). Paired-end cDNA libraries were constructed from 4 μg of total RNA using a KAPA Stranded mRNA-Seq Kit (Kapa Biosystems, Inc., Wilmington, USA) according to the manufacturer's instructions. Samples included biological triplicates of cDNA. The integrity of the libraries was evaluated using a Bioanalyzer 2100 (Agilent Technology Japan, Ltd., Tokyo, Japan). The *de novo* sequencing (76 cycles, paired-end) was performed using Miseq (Illumina, K.K, Tokyo, Japan) at the Center for Bioscience Research and Education, Utsunomiya University.

### De novo transcriptome assembly and functional annotation

The raw sequence reads (76-bp read length) were processed using Trimmomatic (Bolger et al., 2014; Wang et al., 2021) by trimming adapter sequences and low-quality ends (quality score, <15), and discarding the reads shorter than 50 bp. The obtained high-quality reads were further assembled into contigs (unigenes) using Trinity software (Grabherr et al., 2011). The sequences of unigenes were used for BLASTX searches and annotation against the Swiss-Prot protein database to predict biological functions (E-value cut-off was set at 1e-5). KEGG pathway analysis and Gene Ontology (GO) analysis for unigenes were performed using the KEGG automatic annotation server (KAAS) and InterproScan, respectively (Kanehisa and Goto, 2020; Harris et al., 2004).

### Differentially expressed gene analysis

Differentially expressed genes (DEGs) were identified as described previously (Wang et al., 2016). rRNA sequences were excluded from Trinity unigenes by removing matching entries in the rRNA database using the megablast program. High-quality reads derived from each sample were further mapped to rRNA to remove transcripts. Gene expression was calculated using the Fragments Per Million Reads (FPKM) method, and *P*-values were determined by the false discovery rate. DEGs were identified as 2-fold up- or down-regulated with a false discovery rate (FDR) < 0.05 between vegetative cells and cysts in this study.

### Real time PCR analysis

Total RNA extraction, reverse transcription, and Real time PCR reactions were performed according to the previous study (Hakozaki et al., 2023). Total RNAs were isolated from vegetative cells, 2-week-old cysts, and encystment-induced cells as described above. RNA was reverse transcribed using a Transcriptor First Strand cDNA Synthesis Kit (Roche Diagnostics, Mannheim, Germany) according to the manufacturer's instructions. Gene-specific primers were designed using Primer3 software (https://www.bioinformatics.nl/cgi-bin/primer3plus/primer3plus.cgi). The regions of all primers are shown in Table S1. All reactions were performed using the Real time PCR system STEP1 (Thermo Fisher Scientific K.K., Tokyo, Japan) with Power up SYBER (Thermo Fisher Scientific) and three technical replicates. For verification of the RNA-Seq, amplification was performed using the following protocol: 95 °C for 10 min, followed by 40 cycles at 95 °C for 15 s and 60 °C for 1 min. Melt curves were produced according to the manufacturer's instructions. The amplification conditions for analysis of the chitin biosynthesis protein (*CHS5*) gene were described in the manufacturer's instructions. All data were analyzed by the ΔΔct method using Real-Time PCR System Software (Thermo Fisher Scientific). Transcription elongation factor *SPT4* (for Fig. 3C) or the α-tubulin gene (for Fig. 7) of *C. cucullus* (Sogame et al., 2016) were selected as internal control genes.

### Endocyst protein assay

Excystment was induced overnight in cyst samples (10^6^ cells) using protein cut off 1 % rice leaf infusion. Proteins included in the infusion were removed using a 3-kDa cut off ultrafiltration centrifugal filter (Amicon Ultra, Merck Milipore Ltd., Tokyo, Japan). The ectocysts adhering to petri dishes were discarded and the supernatants containing solution soluble endocysts were collected. Before the collection, the excysted cells (vegetative cells) were removed with a 1 μm pore size membrane filter to prevent contamination of proteins of excysted cells, but 3 × 10^3^ cells/100 mL remained in the sample. The endocyst proteins precipitated by 4 times the volume of acetone at -20 °C overnight, centrifuged at 15,000 g at 4 °C for 15 min, air-dried, and the pellet was resuspended in Tris–HCl buffer. The samples were concentrated using a 3-kDa cut filter (Amicon Ultra, Merck Milipore Ltd.) and solubilized with a 4-strength SDS-Page sample buffer. To determine protein contamination by protein cut off 1 % rice leaf infusion and contaminated excysted cells, the same volume of protein cut off 1 % rice leaf infusion and excysted cells in the endocyst samples, were processed in the same way. Additionally, ectocyst samples (vacant cysts after excystment) were prepared to determine protein contamination by ectocysts. The ectocysts corresponding to 10^6^ cells were collected, disrupted using a beads homogenizer and ultrasonic homogenizer, and centrifuged (15,000 x g at 15 min). The pellet was solubilized in SDS-sample buffer and boiled for 3 min.

SDS-PAGE and western blotting on PVDF membranes were performed as described previously (Saito et al., 2020a). Enzymatic digestion of proteins on PVDF membranes was as previously described (Fernandez et al., 1995). Briefly, bands were excised from the membrane, rehydrated in 100 µl ultrapure water, and the pieces of membrane were cut into 1 mm-wide squares. The membrane pieces were placed in a digestion buffer containing 1 % hydrogenated Triton X-100, 10 % acetonitrile, and 100 mM Tris–HCl (pH 8.0) and incubated for 30 min at room temperature. Then, a 1:10 ratio of 0.1 µg/µl of trypsin (Thermo Fisher Scientific) was added to the digestion buffer and the samples were incubated for 24 h at 37 °C. After the incubation, the samples were sonicated twice for 5 min, and 50 µl of digestion buffer was added. The supernatant was transferred to a new tube, and 100 µl 0.1 % trifluoroacetic acid was added. Finally, 2 µl 1 % diisopropylfluorophosphate was added. The extracted peptides were cleaned using Spin Tip columns (C18 resin; Thermo Fisher Scientific) according to the manufacturer's desalted protocol, with a final elution of 20 µl 2 % acetonitrile and 0.1 % formic acid. The concentration of purified peptides was measured using a Nanodrop Spectrophotometer (Thermo Fisher Scientific). After protein extraction and purification, the protein concentrations of the samples ranged from 0.98 to 1.4 mg/ml. Peptides produced by protease digestion were separated with a Thermo UltiMate 3000 UHPLC (Thermo Fisher Scientific) using a 5–80 % effective gradient with mobile phase B (98 % acetone, 0.1 % formic acid) for 60 min. The separated peptides were ionized using a nanoESI source and then subjected to tandem mass spectrometry in a Q-Exactive HF X (Thermo Fisher Scientific). The LC-MS/MS analysis and gene ontology analysis were performed at the Beijing Genomics Institute, China. The results were processed by PEAKS online ver. 10 and obtained peptide sequences were searched in our RNA-seq database (DRR275117-DRR275118.) in which RNA sequences have been translated into amino acid sequences.

## Results

### Bioassay

**Fig. 1:**
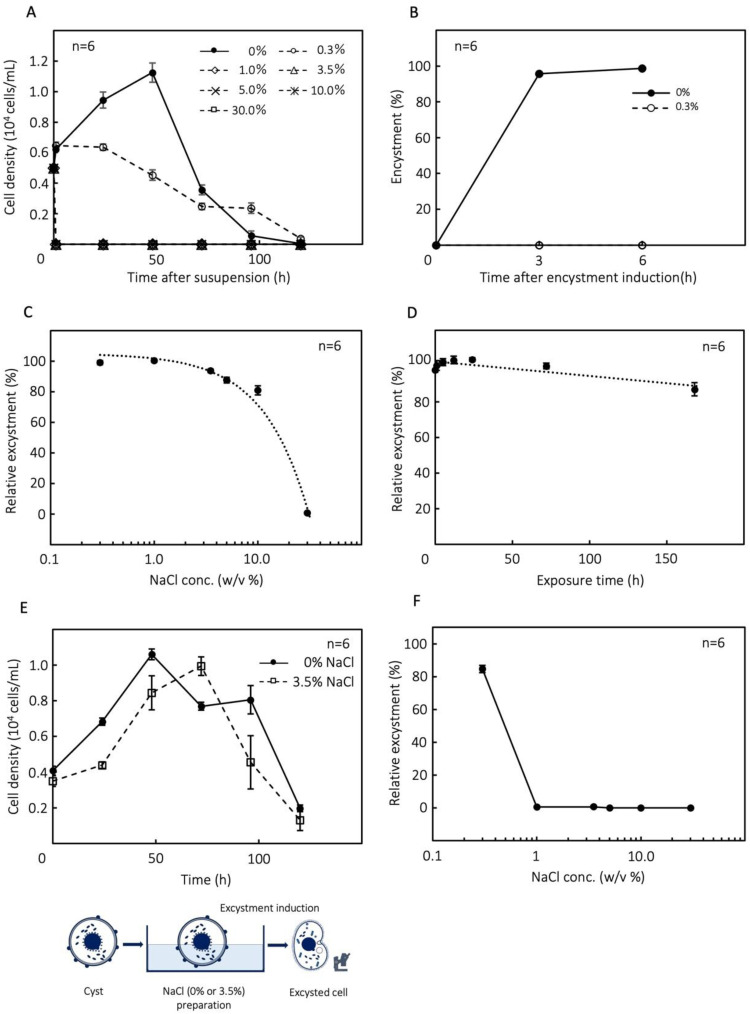
Bioassay of *Colpoda* tolerance to different salinity conditions (0, 0.3, 1, 3.5, 5, 10, 30 % NaCl medium). (A) Vegetative cell tolerance to saline medium. (B) Encystment assay in 0 and 0.3 % saline medium. (C) Excystment assay of *Colpoda* cysts to exposure to saline media for 12 h. (D) Excystment assay of *Colpoda* cysts to 3.5 % saline medium for 0–168 h. (E) Cell proliferation assay of excysted cells from cysts exposed to 0 or 3.5 % NaCl medium. (F) Rate of excystment assay of cysts in different saline media (0, 0.3, 1, 3.5, 5, 10, 30 % NaCl medium).

*C. cucullus* resting cysts were able to survive in 3.5 % NaCl, a salt concentration approximately comparable to seawater, for a week and the cells maintained their proliferative ability. However, vegetative cells died immediately on suspension in high salinity media (1.0–30 %) (Fig. 1A). Vegetative cells survived >24 h in 0.3 % NaCl (Fig. 1A) but could not form cysts (Fig. 1B) or proliferate; they gradually died after >24 h in this medium (Fig. 1A). One-week-old cysts survived in 10 % NaCl for 12 h and showed an excystment rate of 80.9 % (Fig. 1C). A low rate of excystment (0.6 %) was found after 12 h exposure in 30 % NaCl for 12 h. *Colpoda* resting cysts (1-week-old) survived in 3.5 % NaCl for >1 week and showed an excystment rate of 80.1 % (Fig. 1D). After exposure, the resting cysts were able to excyst and proliferate similarly to the control group although at a slower rate (Fig. 1E). Resting cysts treated with 0.3 % NaCl could excyst and revert to the vegetative state under this saline condition, while those suspended in 0.5–30 % NaCl did not show excystment (Fig. 1F).

### Morphological analysis

**Fig. 2:**
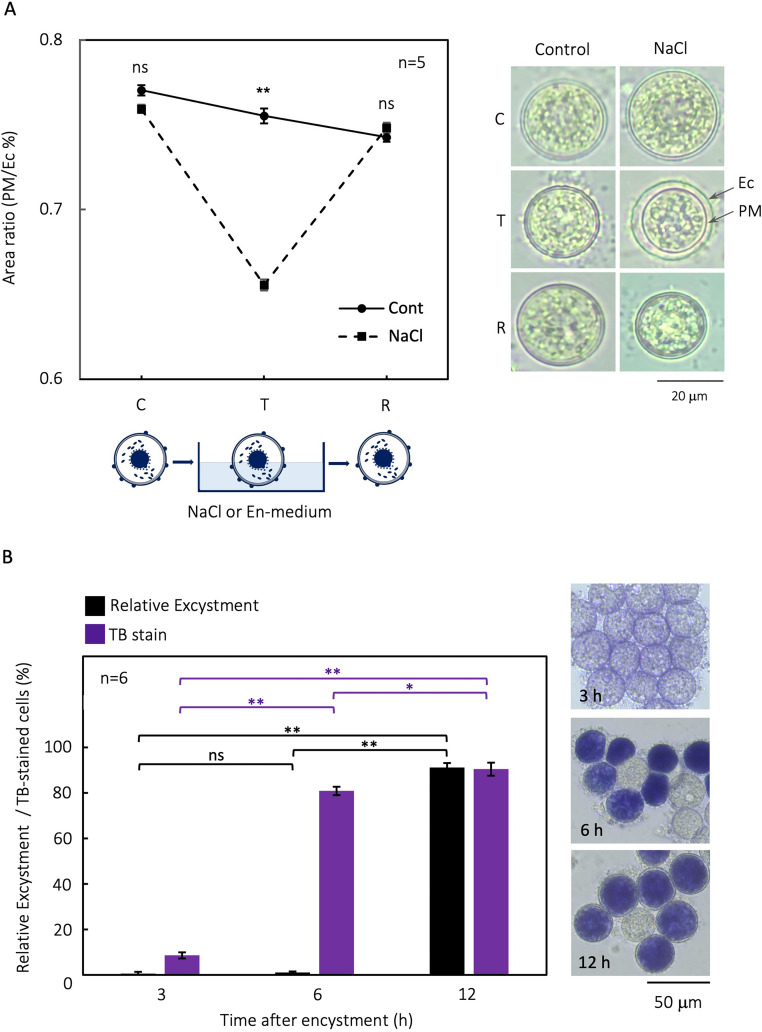
Influence of salinity on *Colpoda cucullus* morphology. (A) Effect of saline medium on the plasma membrane. The horizontal axis represents three experimental status: 1-week-old cysts ‘C’, 1-week-old cysts treated with En-medium as control samples (‘Cont’) or 3.5 % NaCl as experimental samples (‘NaCl’) for 1 week ‘T’, and resuspended in En-medium after the preparation ‘R’. The vertical axis represents the area ratio [area surrounded by PM / area surrounded by Ec]. (B) Micrographs show TB staining at 3, 6, and 12 h after the onset of encystment induction. The rate of TB staining and relative excystment rate at 3, 6, and 12 h after induction of encystment in cysts suspended in 3.5 % NaCl for 1 h. * *p* < 0.05; ** *p* < 0.01; ns, no significant difference.

Vegetative cells were impaired immediately after being suspended in a high-salinity medium, but cysts remained viable. This indicate that the function of plasma membrane of resting cysts is maintained even under salinity stress. When 1-week-old cysts (Fig. 2A ‘C’) were treated En-medium containing 3.5 % NaCl for 1 week (Fig. 2A ‘T’), there was clear shrinkage inside the cyst; however, this mostly recovered when the cysts were resuspended in the En-medium (Fig. 2A ‘R’). Hence, we concluded that the plasma membrane maintained its functionality even when exposed to high salinity, and protected the cell from its adverse effects.

TB staining showed that the endocyst was not involved in the high salinity tolerance of resting cysts. At 3 h after the induction of encystment, <10 % of the cells were TB-stained; by contrast, over 80 % of cells were TB-stained at 6 h and 12 h (Fig. 2B ‘TB stain’). The relative excystment rates after treated with high salinity (3.5 % NaCl for 1 h) were not significant at 3 h and 6 h (≤1 %, respectively) but were significantly elevated at 12 h (>90 %; Fig. 2B ‘Relative excystment’). Overall, there was no correlation between salinity tolerance and the rate of endocyst formation.

### Transcriptomic profiles of vegetative cells and cysts

Paired-end sequences (2 × 75 bp in length) from *C. cucullus* vegetative cells and cysts were generated by Miseq, which produced 13,518,200 reads and 12,733,840 reads, respectively (Table S2). The de novo assembly results are summarized in Table S3. In total, we obtained 73,011 (N50 length = 1443 bp) unigenes in *C. cucullus* (Table S3). The total length was 65,639,138 bp, and the maximum sequence length was 17,589 bp; the minimum sequence length was 201 bp (Table S3). We found that the highest number of unigenes was represented in groups 201–300, and the lowest in group 1901–2000 (Fig. S1). All the transcriptome data are provided in Table S4 and the sequence reads have been deposited in the DDBJ Sequence Read Archive (DRA)/SRA under the accession numbers DRR275117-DRR275118.

### MA and volcano plot analysis

**Fig. 3:**
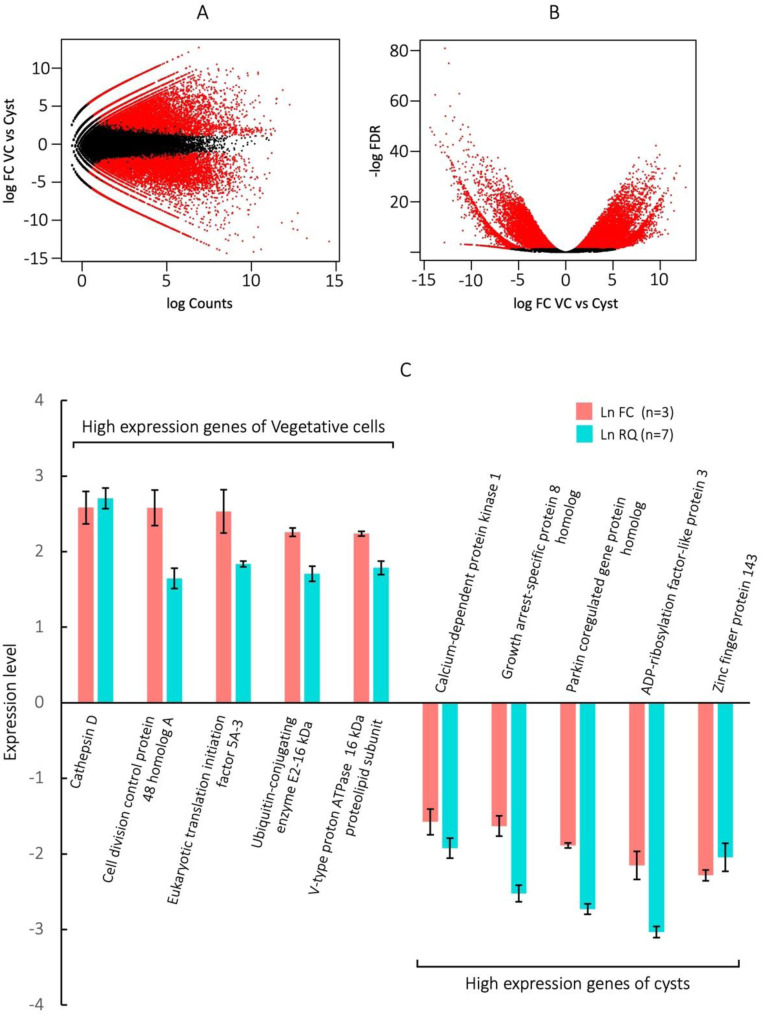
MA plot, Volcano plot, and verification of the RNA-Seq data by qRT-PCR analysis in *C. cucullus* vegetative cells and cysts. (A) MA plot of DEGs. The x-axis represents the log2 transformed mean expression level of unigenes. The y-axis represents the log2 transformed fold change in vegetative cells vs cysts. The positive or negative value indicates vegetative cell-specific or cyst-specific, respectively. (B) Volcano plot of DEGs. The x-axis represents the log2 transformed fold change in vegetative cells vs cysts. The positive or negative value indicates vegetative cell-specific or cyst-specific, respectively. The y-axis represents the log2 transformed fold change. *P* < 0.05, FC >1 genes are indicated by red points. (C) Verification of the RNA-Seq (Ln FC) data by qRT-PCR analysis (relative gene expression quantity; Ln RQ). Five genes with high expression in vegetative cells and cysts were selected. FC (*n* = 3) corresponds to the expression level of the transcriptomic analysis and RQ (*n* = 7) is that of qRT-PCR.

We used an MA plot to compare the log_2_ fold-changes (Log_2_ FC) of unigenes in vegetative cells and cysts and mean expression (log counts) (Fig. 3A). Overall, 14,056 differentially expressed unigenes (DEGs) were identified in vegetative cells (FDR <0.05, Log_2_FC >1) and 11,258 in cysts (FDR <0.05, -Log_2_FC >1); these are shown as red points in the MA plot (Fig. 3A). A comparison of P-value and log fold-change between vegetative cells and cysts is shown in the volcano plot (Fig. 3B). DEGs in the vegetative cells (FDR <0.05, Log_2_FC >1) and cysts (FDR <0.05, -Log_2_FC >1) are shown as red points.

### Selected DEGs analysis

The expression level of selected genes: related to ribosome biogenesis and protein production, AMP-activated protein kinase (*AMPK*), Extracellular signal-regulated kinase 1/2 (*ERK1/2*), Autophagy-related protein 8 (*ATG8*), Serine/threonine-protein kinase (*SGK1*), and Elongation factor 1-alpha (*EF-1α*) were summarized in Table S5.

### Verification of RNA-Seq data

The expression levels of five up-regulated genes in vegetative cells and cysts were verified by real-time PCR. The mRNA expression levels of these genes were consistent with the transcriptomic data (Fig. 3C). Transcription elongation factor *SPT4* was used as the internal control as its expression is similar in vegetative cells and cysts. The names of the selected genes are given in Fig. 3C and their gene IDs and amplification primers are shown in table S1.

### Functional annotation of DEGs and GO enrichment analysis

All unigenes were searched against the Swiss-Prot database; the 23,265 unigenes were identified. Unigenes that were over 2-fold up- or downregulated were isolated: 14,056 genes were expressed significantly higher in vegetative cells, whereas 11,258 genes were expressed significantly higher in resting cysts (Table S4).

**Fig. 4:**
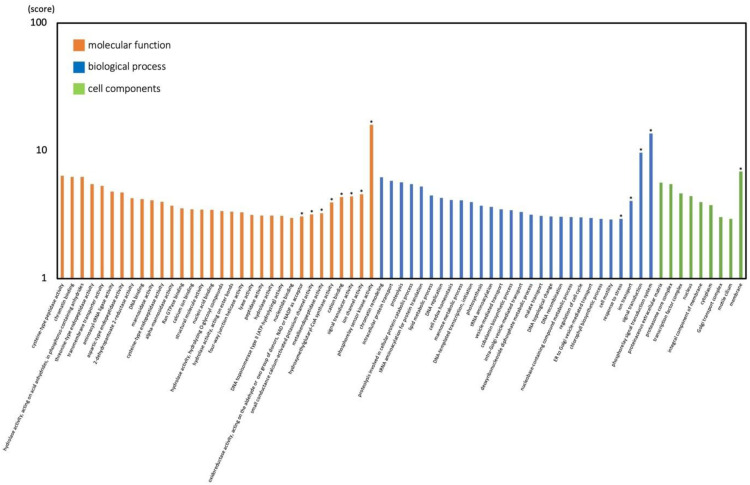
GO functional classification of differentially expressed unigenes. Z score of GO terms is shown as the y-axis. Asterisks indicate cysts. GO functional classification of differentially expressed unigenes. Z score of GO terms is shown as the y-axis. Asterisks indicate cysts.

**Fig. 5:**
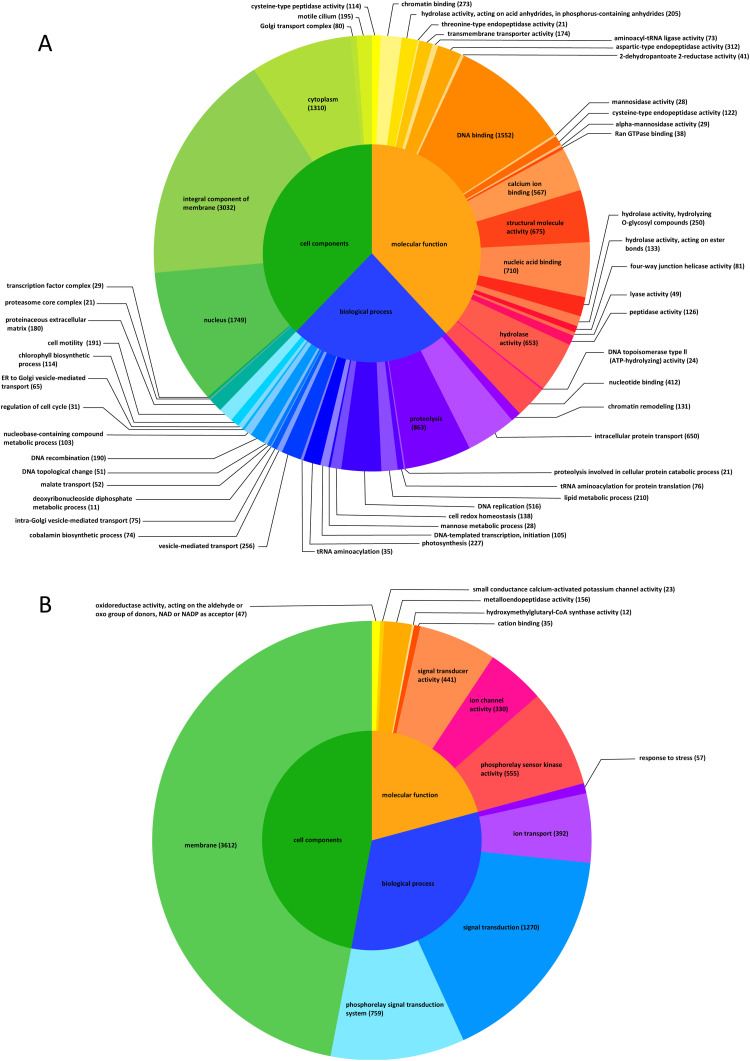
Gene ontology enrichment analysis of the DEGs in vegetative cells (A) and cysts (B).

We performed GO enrichment analysis of vegetative cells and cysts and characterized the 98,560 unigenes as 897 GO terms. These GO terms could be divided into 374 categories of Biological Process, 383 categories of Molecular Function, and 140 categories of Cell Components (Table S6). Expression levels were significantly different between vegetative cells and cysts for 28 Biological Process categories with 6691 unigenes, 32 Molecular Function categories with 5215 unigenes, and 9 Cell Components categories with 498 unigenes. In vegetative cells, unigenes of GO category ‘cysteine-type peptidase activity’ showed the greatest upregulation, followed by ‘chromatin binding’, and ‘hydrolase activity, acting on acid anhydrides, in phosphorus-containing anhydrides’ (Fig. 4). In cysts, unigenes of the GO category ‘phosphorelay sensor kinase activity’ showed greatest upregulation, followed by ‘phosphorelay signal transduction system’ and ‘signal transduction’ (Fig. 4).In vegetative cells (Fig. 5A), the number of unigenes in the GO category ‘integral component of membrane’ was the largest and similarly for the GO Cell Components type. The GO ‘nucleus’ and ‘DNA binding’ categories also contained many of the unigenes: the number of unigenes in ‘DNA binding’ and ‘proteolysis’ were the largest in Molecular Function and Biological Process, respectively. In resting cysts (Fig. 5B), overall, unigenes of Molecular Function and Biological Process were downregulated, whereas those in the ‘membrane’ category of Cell Components were upregulated. The category of Biological Process contained a large number of the identified DEGs, although the category was simplified. The GO ‘membrane’ category was the largest and all of them are included in the GO category Cell Components which is 47 % of all unigenes in resting cysts. The GO ‘signal transduction’ and ‘phosphorelay signal transduction system’ categories also included many unigenes: ‘signal transduction’ and ‘phosphorelay sensor kinase activity’ were the largest categories in Biological Process and Molecular Function, respectively. In addition, the expression of genes related to stress responses and oxidoreductase activity showed upregulation, while other metabolic processes displayed down-regulation.

### Analysis of the cyst wall: ectocyst components and endocyst proteins

**Fig. 6:**
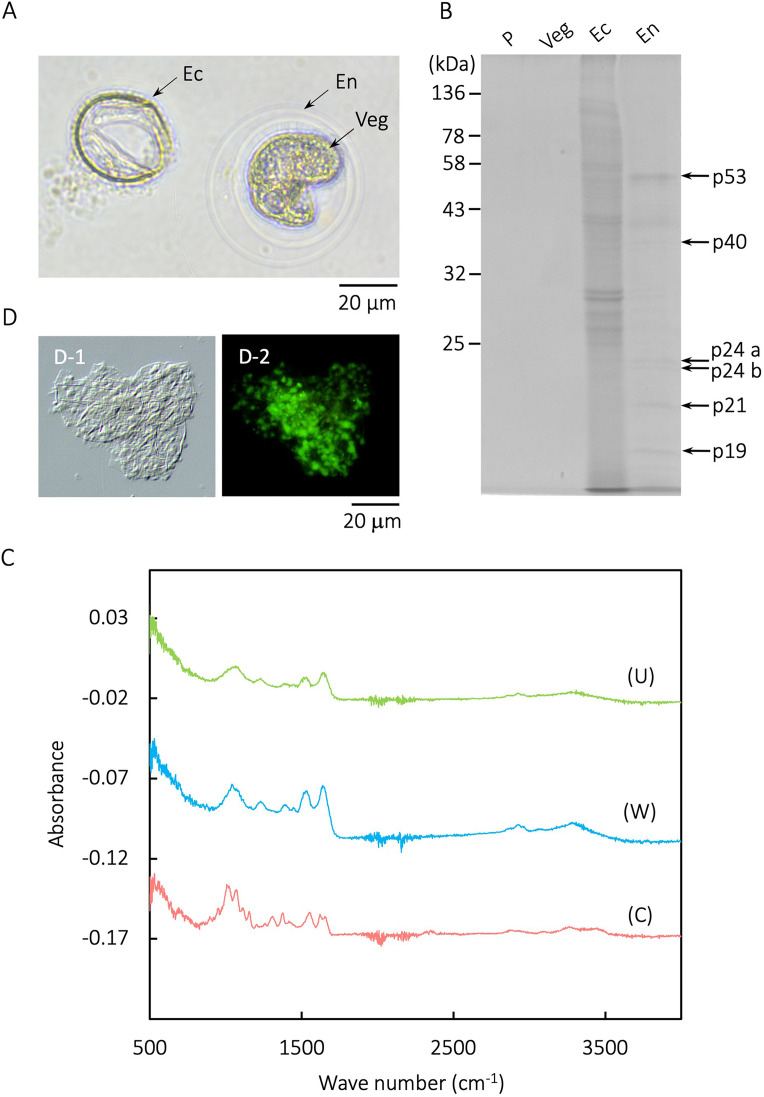
Analysis of cyst wall (endocyst and ectocyst) components. (A) Light microscope images of an excysting vegetative cell (Veg) and the main two layers of cyst wall: endocyst (En) and ectocysts (Ec). (B) Identification of endocyst-specific proteins. SDS-PAGE of proteins from endocysts (En), ectocysts (Ec), and vegetative cells (which seem to show some contamination by the endocyst sample (Veg), and protein cut off 1 % rice leaf infusion (P). Endocyst-specific protein bands (p53, 40, 24a/b, 21, and 19) were identified by LC-MS/MS and the results are shown in Table 1. (C) ATR-IR spectra of an unwashed ectocyst sample (U), an ethanol-washed ectocyst sample (W), and pure chitin sample (C). An overview of absorbance peaks is shown in Table 2. (D) Lectin staining (FITC-conjugated WGA) of an ectocyst sample by Differential Interference Contrast (DIC) microscopy (D-1) and fluorescence microscopy (D-2). The bar represents 20 μm.

An excysting cell and the two main layers of the cyst wall are shown in Fig. 6A. As our analyses have indicated that cyst wall components are likely to be involved in salinity tolerance, we sought to identify them. Previous studies have revealed many of the protein components of the cyst wall (ectocyst) (Izquierdo et al., 1999; Izquierdo et al., 2000; Chessa et al., 2002) and the presence of elongation factor Tu (EF-Tu) in lepidosomes (Funadani et al., 2016) in the genus *Colpoda*. However, there is no information to date on purified endocysts. Here, endocyst proteins were purified and electrophoresed by SDS-PAGE (Fig. 6B ‘En’); the figure also shows proteins from protein cut off 1 % rice leaf infusion (Fig. 6B ‘P’), vegetative cells (Fig. 6B ‘Veg’), and ectocysts (Fig. 6B ‘Ec’). SDS-PAGE indicated that the endocyst sample was unaffected by protein cut off 1 % rice leaf infusion and vegetative cells. The endocyst-specific protein bands (p53, p40, p24a, p24b, p21, and p19), which were not present in ectocysts, were analyzed by LC-MS/MS and a summary of the results is shown in Table 1. Bands p53, p40, and p21 contained predicted protein homologous to actin; p53 also contained elongation factor 1α. In addition, some enzymes, membrane proteins, and transmembrane proteins were detected (Table 1).

**Table 1:** Identification of endocyst specific protein bands (p53, 40, 24a, 24b, 21, and 19) by LC-MS/MS analysis.

Protein band	Matched Peptides	E value	Per.Ident (%)	TRINITY_ID	protein name
p53	4	0.0	97.60	TRINITY_DN31814_c0_g2_i1.p1	predicted protein [Hordeum vulgare subsp. vulgare]
	3	2E-121	66.53	TRINITY_DN22038_c0_g1_i1.p1	phosphoglycerate mutase, related [Neospora caninum Liverpool]
	2	3E-162	55.87	TRINITY_DN29808_c0_g1_i1.p1	L‑serine ammonia-lyase [Hahella sp. CCB-MM4]
	2	0.0	79.77	TRINITY_DN30384_c0_g1_i1.p1	atp synthase alpha subunit [Stylonychia lemnae]
	2	0.0	78.10	TRINITY_DN31063_c1_g2_i1.p1	elongation factor 1 alpha, partial [Arachnula sp. CL12]
	2	6E-47	100.00	TRINITY_DN33760_c0_g1_i1.p1	nitrogen regulatory protein P-II 1 [Agrobacterium tumefaciens]
	2	6E-45	85.57	TRINITY_DN7118_c0_g1_i1.p1	AAA ATPase domain-containing protein [Dictyostelium discoideum AX4]
p40	6	0.0	79.77	TRINITY_DN30384_c0_g1_i1.p1	atp synthase alpha subunit [Stylonychia lemnae]
	3	2E-121	66.53	TRINITY_DN22038_c0_g1_i1.p1	phosphoglycerate mutase, related [Neospora caninum Liverpool]
	3	3E-172	58.30	TRINITY_DN29808_c0_g2_i1.p1	L‑serine ammonia-lyase [Hahella sp. CCB-MM4]
	3	5E-48	97.56	TRINITY_DN30698_c0_g2_i10.p1	histone H4-like [Drosophila rhopaloa]
	3	0.0	97.60	TRINITY_DN31814_c0_g2_i1.p1	predicted protein [Hordeum vulgare subsp. vulgare]
p24a	5	0.0	79.77	TRINITY_DN30384_c0_g1_i1.p1	atp synthase alpha subunit [Stylonychia lemnae]
	2	3E-162	55.87	TRINITY_DN29808_c0_g1_i1.p1	L‑serine ammonia-lyase [Hahella sp. CCB-MM4]
	2	6E-45	85.57	TRINITY_DN7118_c0_g1_i1.p1	AAA ATPase domain-containing protein [Dictyostelium discoideum AX4]
p24b	4	3E-173	71.34	TRINITY_DN28259_c0_g1_i1.p1	type I glyceraldehyde-3-phosphate dehydrogenase [Yeguia hominis]
	3	0.0	79.77	TRINITY_DN30384_c0_g1_i1.p1	atp synthase alpha subunit [Stylonychia lemnae]
	2	6E-47	100.00	TRINITY_DN33760_c0_g1_i1.p1	nitrogen regulatory protein P-II 1 [Agrobacterium tumefaciens]
	2	6E-45	85.57	TRINITY_DN7118_c0_g1_i1.p1	AAA ATPase domain-containing protein [Dictyostelium discoideum AX4]
	2	3E-157	57.77	TRINITY_DN25718_c0_g1_i1.p1	L‑serine ammonia-lyase [Noviherbaspirillum sp. UKPF54]
p21	9	5E-48	97.56	TRINITY_DN30698_c0_g2_i10.p1	histone H4-like [Drosophila rhopaloa]
	4	0.0	79.77	TRINITY_DN30384_c0_g1_i1.p1	atp synthase alpha subunit [Stylonychia lemnae]
	4	0.0	97.60	TRINITY_DN31814_c0_g2_i1.p1	predicted protein [Hordeum vulgare subsp. vulgare]
	3	3E-173	71.34	TRINITY_DN28259_c0_g1_i1.p1	type I glyceraldehyde-3-phosphate dehydrogenase [Yeguia hominis]
	3	3E-172	58.30	TRINITY_DN29808_c0_g2_i1.p1	L‑serine ammonia-lyase [Hahella sp. CCB-MM4]
	2	6E-47	100.00	TRINITY_DN33760_c0_g1_i1.p1	nitrogen regulatory protein P-II 1 [Agrobacterium tumefaciens]
	2	6E-45	85.57	TRINITY_DN7118_c0_g1_i1.p1	AAA ATPase domain-containing protein [Dictyostelium discoideum AX4]
	2	8E-61	94.34	TRINITY_DN20942_c0_g1_i1.p1	Histone-fold [Pseudocohnilembus persalinus]
p19	4	3E-173	71.34	TRINITY_DN28259_c0_g1_i1.p1	type I glyceraldehyde-3-phosphate dehydrogenase [Yeguia hominis]
	2	2E-121	66.53	TRINITY_DN22038_c0_g1_i1.p1	phosphoglycerate mutase, related [Neospora caninum Liverpool]
	2	3E-172	58.30	TRINITY_DN29808_c0_g2_i1.p1	L‑serine ammonia-lyase [Hahella sp. CCB-MM4]
	2	0.0	79.77	TRINITY_DN30384_c0_g1_i1.p1	atp synthase alpha subunit [Stylonychia lemnae]
	2	6E-45	85.57	TRINITY_DN7118_c0_g1_i1.p1	AAA ATPase domain-containing protein [Dictyostelium discoideum AX4]

The spectra obtained in an ATR-IR analysis indicated that the cyst wall flake contained chitin. The main peaks and their attribution are summarized in Table 2. For ectocysts, the spectra from the ATR-IR analysis were like that of α-chitin reported by Van de Velde et al. (2004) and chitin standard (Fig. 6C, Table 2, except for the O–H stretching region (3100–3600 cm^-1^). The two broad OH peaks of chitin (3433 cm^-1^, 3252 cm^-1^) were present as a single broad peak (3272 or 3279 cm^-1^) in the unwashed and ethanol-washed ectocyst samples (Fig. 6C, Table 2). There was an additional small difference between the chitin samples and the ectocyst samples under 3100 cm^-1^.

**Table 2:** Overview of absorbance peaks of ATR-IR spectra of cyst wall and α-chitin samples. The spectra peaks of unwashed ectocyst sample 'U', ethanol washed ectocyst sample 'W', pure chitin sample 'C'and that by Van de Velde and Klekens, 2004 are shown.

	Cyst wall		chitin	α-chitin
Attribution	(U) unwash	(W) washed	(C) This work	Van de Velde and Kiekens, 2004
O–H stretching	Shoulder	Shoulder	3433	3450
N–H stretching	3272	3279	3252	3265
C–H stretching	2916	2918	2860	2878
C = O stretching	Shoulder	Shoulder	1650	1655–61
	1638	1637	1619	1625
	1511	1524	1545	1554
C–N stretching	1385	1389	1371	1380
O–H bending	shoulder	Shoulder	1298	1320
C–H bending	1225	1223	–	1205
	1054	1043	1064	1076
	Shoulder	Shoulder	1006	1026

The results of the lectin (WGA) staining supported those of the ATR-IR analysis. Ectocyst samples (Fig. 6D-1) were stained with FITC-labeled WGA, a lectin that binds with chitin (Kristiansen et al., 1999); a FITC signal was detected in the ectocysts (Fig. 6D-2), indicating the presence of chitin in the ectocyst.

**Fig. 7:**
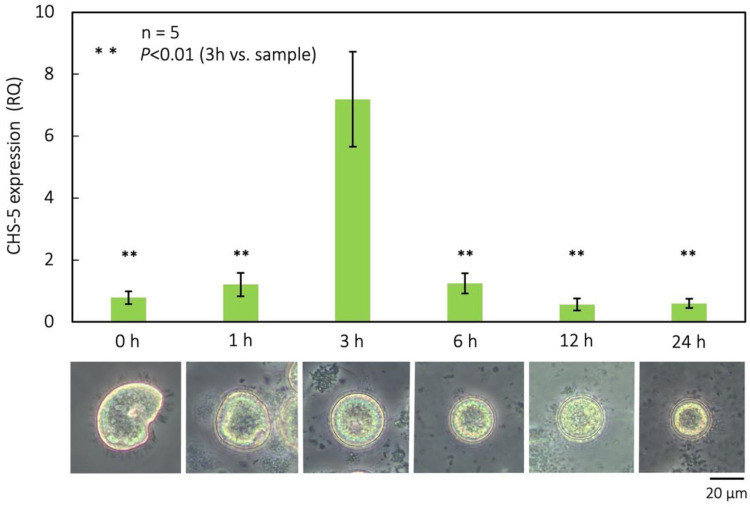
Morphological changes in the encystation process of *C. cucullus* and qRT-PCR analysis of expression of *CHS5* gene during this process. Double asterisks represent the significant differences (*p* < 0.01) between each samples and the 3 h sample.

*CHS5* is involved in chitin synthesis in *Saccharomyces cerevisiae* (Santos et al., 1997) and has been shown to have a temporary increase in expression during ectocyst synthesis (Fig. 7). The ectocyst is gradually formed during encystation and can be observed at 3 h after encystment induction (Fig. 7 '3 h'). We performed real-time PCR analysis and showed that expression of the *CHS5* gene was increased temporarily at 3 h after the onset of encystment in *Colpoda*.

## Discussion

The morphological analysis showed plasma membrane of resting cysts was maintained even in high salinity condition. Gene ontology analysis revealed that properties of the membrane including ion channel activity and ion transport were changed considerably during encystation suggested reconstruction of plasma membrane. Hence, salinity tolerance in *Colpoda* resting cysts is involved in reconstruction of plasma membrane in encystation, and these changes probably enable it to maintain its structure in high salinity conditions. The plasma membrane of resting cysts, which was exposed to high salinity retained its functionality, however, this study was unable to clarify presence or absence of damages and the mechanism for the reconstruction. The transcriptomes did not analyze along a salinity gradient, the changes in gene expression may reflect many environmental factors related to encystment, rather than the indications of only salinity effect, or salinity specific. In addition, such membrane function proteins are not only located in the plasma membrane but also in membrane of intracellular organelles, such as mitochondria. Hence, they possibly affect membrane structures other than the plasma membrane, for example blocking the mitochondrial membrane potential during encystation (Funatani et al., 2010; Sogame et al., 2014a; Hakozaki et al., 2023).

The comparison of transcriptomes showed upregulation of genes related to stress responses and oxidoreductase activity in resting cysts. One probable strategy to protect cells from stress is the involvement of FOXO members (signaling pathway), which was proposed by Pan et al. (2019). Our results of upregulation of Autophagy-related protein 8 (*ATG8*) whereas downregulation of Serine/threonine-protein kinase (*SGK1*) also suggest a contribution of FOXO signaling pathway. FOXO members are known to contribute to protecting cells against oxidative stress (Essers et al., 2004) and the carbonylated proteins were rescued then cell damages were repaired in *Colpoda* resting cysts (Sogame et al., 2019; Saito et al., 2020a).

Differential gene expression during the early stage of encystment has been shown previously in transcriptomic studies of *Colpoda aspera* (Jiang et al., 2019) and *Pseudourostyla cristata* (Pan et al., 2019); these observations demonstrated the presence of an early signaling pathway for encystment as had been suggested earlier (Matsuoka et al., 2009; Matsuoka, 2021). This signaling pathway is involved in numerous intracellular events, e.g., it is linked to autophagy through AMPK activation and regulation of cell cycle through Extracellular signal-regulated kinase (ERK) (Chambard et al., 2007) during resting cyst formation (Jiang et al., 2019). Our transcriptome data confirmed the enhancement of AMPK activation and upregulation of *ATG8* and *ERK1/2* genes, in resting cysts as described by Jiang et al. (2019). During resting cyst formation, the expression level of many proteins is altered and the amount of intracellular proteins is reduced (Sogame et al., 2014a) via regulation of protein synthesis and degradation. Jiang et al. (2019) and Griavard et al. (2008) described the downregulation of genes related to the ribosome and protein biogenesis and Pan et al. (2019) indicated the protein degradation via the ubiquitin-proteasome system in resting cyst formation. The system is activated not only during dormant cyst formation but also during starvation in *Tetrehymena* (Miao et al., 2009), suggesting that it is a broadly adaptive stress response. Our transcriptome data confirmed their findings. Encystment in *C. cucullus* is controlled by a complex pattern of gene expression and can respond to many extracellular stresses. Differential gene expressions lead to morphological changes, e.g., the characteristic broad bean cell shape gradually becomes spherical (Asami et al., 2010), a cyst wall is formed, and cilia and mitochondria are digested (Funatani et al., 2010).

Chitin is shown to be a main component of the ectocyst which may be synthesized and act as a chitinous biological armor during the *C. cucullus* encystation process. Carbohydrates such as cellulose in plants and chitin in invertebrates coat cell surfaces. The cyst walls of ciliates also contain carbohydrates (Gutiérrez et al., 2003), and the presence of chitin has been inferred in some ciliates following chitinase digestion (Gutiérrez et al., 2003; Bussers et al., 1974; Bussers et al., 1976). However, *Colpoda* species (*C. cucullus, C. steinii*) have been reported to test negative for chitinase digestion, indicating the absence of chitin (Gutiérrez et al., 2003; Izquierdo et al., 1999; Bussers et al., 1976). On the other hand, we confirmed the presence of chitin in ectocysts by ATR-IR analysis and lectin staining in this study. ATR-IR spectra of *Colpoda* ectocysts were like that of α-chitin reported by Van de Velde et al. (2004) and chitin standard, however, spectra of O–H stretching region (3100–3600 cm^-1^) and under 3100 cm^-1^ show some differences between *Colpoda* ectocyst samples and chitin. The difference may be due to interaction between chitin and other components in the cyst wall as the OH stretching modes might be significantly influenced by hydrogen bonding with other components such as oil and proteins. It may be explained by the effect of other components such as acetylation or crystallinity. We washed ectocyst sample with ethanol to remove these components but no change was observed except for the reduction of some noise. In previous studies, it is possible that the enzymatic activity of chitinase was inhibited in some manner, possibly involving highly acidic proteins (Gutiérrez et al., 2003). Our method is completely unaffected by that. Although chitin has been found in the cyst walls of many ciliates, chitin synthase activity has not yet been reported (Gutiérrez et al., 2003). The *CHS5* gene is not the chitin synthase itself but is considered to be involved in chitin synthesis as the knockout of *CHS5* expression inhibits chitin synthesis in yeast (Santos et al., 1997). Hence, the result that activation of *CHS5* expression coincided with the formation of ectocysts supports the interpretation that chitin is a component of ectocysts.

In addition to the ectocyst as a chitinous biological armor, the inner endocyst is likely to serve as protective materials by shielding the cell from dehydration. Actin and EF-1α were contained in protein bands of endocyst components. Actin is likely to form filamentous actin (F-actin) in ectocyst and endocyst cell walls and lepidosomes can be stained with Phalloidin (Sogame et al., 2020). However, it is possible that this staining may be partially attributable to the autofluorescence of the endocyst layer (Sogame et al., 2020). The presence of actin in endocyst is unlikely to be a contaminant from ectocysts and/or lepidosomes, as actin has not been detected in samples of ectocysts. Most probably, the actin originated from endocysts. However, it is difficult to eliminate contamination of intracellular actin with the purification method used for endocysts in this study. A similar limitation may apply to other detected proteins, for example, the histones and membrane proteins such as ATP synthase α subunit that were detected in some protein bands of endocysts. In contrast, it is possible that some proteins of the cell are included and that they are materials used to construct the endocyst since endocyst layers are formed by the exocytosis of short filamentous precursor materials (Matsuoka, 2021; Funatani et al., 2010). The fact that endocyst layers are stained with TB (Sogame et al., 2011c; Funatani et al., 2010) indicates that they include mucopolysaccharides. The gene expression level of EF-1α and its protein level (Sogame et al., 2012a) were elevated during encystment and may contribute to endocyst formation by polymerization of actin (Kurasawa et al., 1996). On the other hand, the level of EF-1α is reduced within 1 h of excystment induction (Sogame et al., 2013). This process is likely dependent on actin depolarization. The actin identified in this study had a molecular weight different from the expected 42 kDa. This is probably due to the influence of post-translational modification, such as acetylation and phosphorylation, as has been observed in *Entamoeba histolytica* (Hernández-Cuevas et al., 2019), or fragmentation due to the use of trypsin. Various molecular weights of actin were detected from Hela cell protein samples (Thiede et al., 2013). Other membrane proteins may also function as a second protective material by confining water or acting like a polymer absorber to maintain humidity and protect against desiccation.

**Fig. 8:**
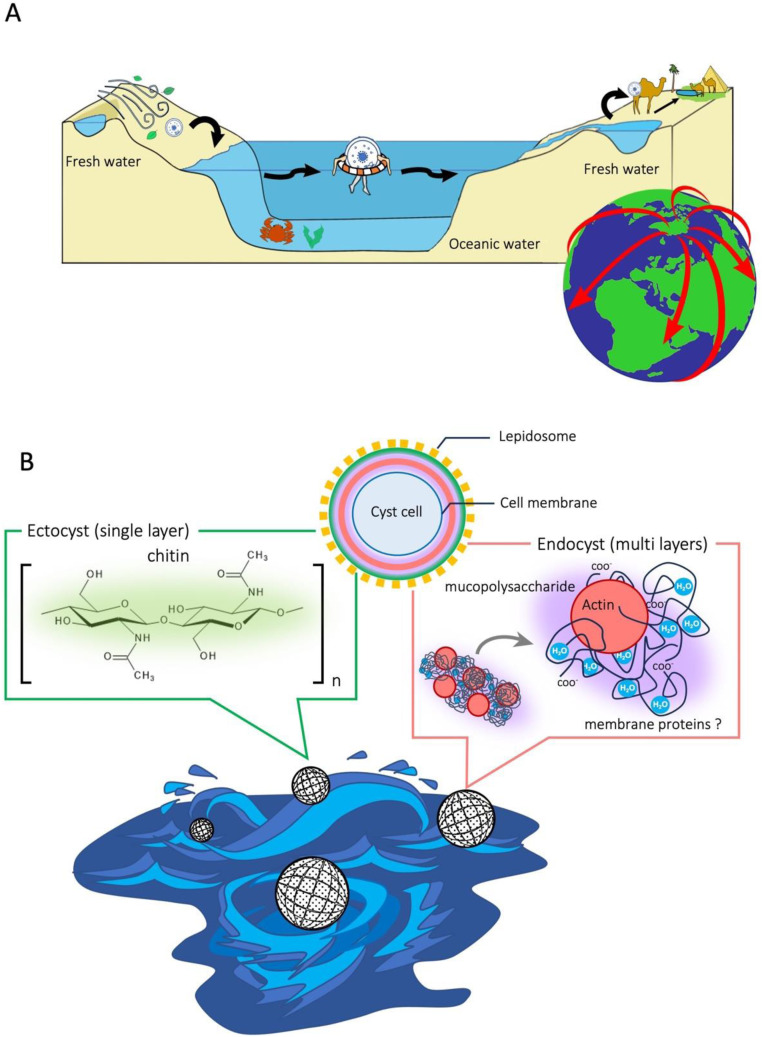
Illustration of our proposed model for oceanic dispersal of freshwater protists (A) and possible protection from mechanical stress by the chitinous ectocyst (B). (A) Freshwater ciliates such as *Colpoda* live in a temporary aqueous environment and they form resting cysts when the water disappears due to desiccation. Cysts may be transferred by wind, animals or human activity. Cysts that have accidentally been transported to the ocean get a chance to travel. (B) During their ocean travel, the cysts face stormy seas and mechanical stresses; however, the chitinous ectocyst protects the cysts from the stresses.

This study also suggests a possible scenario's oceanic dispersal of freshwater protists as shown in Fig. 8. It is possible that they could cross oceans as floating cysts (Fig. 8A), being protected by a cyst wall consisting of a chitinous outermost layer (ectocyst) and several inner layers (endocyst) of actin-like proteins with mucopolysaccharides (Fig. 8B). The cysts would be passively transferred by oceanic currents which enable long-distance transportation (Villa Martín et al., 2020). This oceanic dispersal might be complemented by other means such as transport by bugs, birds, or human activities (Foissner, 2006, 2011; Bharti et al., 2020). While they are sailing, the chitinous ectocyst protects against physical shocks including strong water currents and bubbles (Fig. 8B). It might be feasible to search for freshwater protists ‘sailing’ across the ocean through environmental DNA analysis; this is a topic for further research.

Protists, including ciliates, are ubiquitously abundant in both marine environments with high osmotic pressure and hypotonic, freshwater environments. These habitats are completely different and the species have evolved in parallel. Marine protists accumulate osmoregulatory solutes (Welsh, 2020), while freshwater protists have evolved contractile vacuoles (Hoef-Emden et al., 2014). Despite the parallel evolution, resting cyst formation could enable freshwater ciliates (*Colpoda*) to survive oceanic dispersal due to their salinity tolerance. The resting cyst formations a common strategy for micro-organisms to survive in severe environments (Li et al., 2022). It give a chance not only to survive in unfavorable environments but also to disperse.

## Funding

This research was financially supported by JSPS KAKENHI (19K16193, 22K06326, 23H02702, S23134), and the Ministry of Education, Culture, Sports, Science and Technology, Japan (MEXT) to a project on Joint Research Center– Leading Academia in Marine and Environment Pollution Research (LaMer).

## CRediT authorship contribution statement

**Ryota Saito:** Data curation, Formal analysis, Investigation, Validation, Visualization, Writing – original draft, Writing – review & editing. **Hiroki Yamanobe:** Data curation, Formal analysis, Investigation, Validation, Visualization, Writing – review & editing. **Kazuma Yabuki:** Data curation, Formal analysis, Investigation, Validation, Writing – review & editing. **Tomohiro Suzuki:** Data curation, Formal analysis, Investigation, Validation, Visualization, Writing – original draft, Writing – review & editing. **Takeru Saito:** Data curation, Formal analysis, Investigation, Validation, Writing – review & editing. **Shuntaro Hakozaki:** Data curation, Formal analysis, Investigation, Validation, Visualization, Writing – review & editing. **Manfred Wanner:** Writing – original draft, Writing – review & editing. **Ryota Koizumi:** Data curation, Formal analysis, Investigation, Validation, Writing – review & editing. **Tatsuya Sakai:** Data curation, Formal analysis, Investigation, Validation, Writing – review & editing. **Maribet Gamboa:** Data curation, Formal analysis, Investigation, Validation, Writing – review & editing. **Toshihiko Tanaka:** Data curation, Formal analysis, Investigation, Validation, Visualization, Writing – original draft, Writing – review & editing. **Akiko Ono:** Data curation, Formal analysis, Investigation, Validation, Writing – review & editing. **Hoa Thanh Nguyen:** Data curation, Formal analysis, Investigation, Validation, Writing – review & editing. **Yuta Saito:** Data curation, Formal analysis, Investigation, Validation, Writing – review & editing. **Tetsuya Aoyama:** Data curation, Formal analysis, Investigation, Validation, Writing – review & editing. **Katsuhiko Kojima:** Formal analysis, Writing – review & editing. **Futoshi Suizu:** Funding acquisition, Writing – review & editing. **Kozo Watanabe:** Funding acquisition, Writing – review & editing. **Yoichiro Sogame:** Conceptualization, Data curation, Formal analysis, Investigation, Funding acquisition, Supervision, Validation, Visualization, Writing – original draft, Writing – review & editing.

## Declaration of competing interest

The authors declare that they have no known competing financial interests or personal relationships that could have appeared to influence the work reported in this paper.

## Data Availability

Data will be made available on request.

## Appendix Supplementary materials

Image, application 1

Image, application 2

Image, application 3

Image, application 4
