# Supplementary material for: Salinity tolerance in resting cysts of colpodid ciliates: Comparative transcriptomics analysis and chemical analysis of cyst walls to investigate their tolerance capability
Source: Curr Res Microb Sci. 2025 Mar 7;8:100371. doi: 10.1016/j.crmicr.2025.100371 (PMC11952024; doi:10.1016/j.crmicr.2025.100371)
Supplement: Supplementary file 1 [file mmc1.docx]

**Supplementary Materials**

**Salinity tolerance in resting cysts of colpodid ciliates: comparative transcriptomics analysis and chemical analysis of cyst wall towards investigation its tolerance capability**

Ryota Saito^†1,2^, Hiroki Yamanobe^1,3^, Kazuma Yabuki^1^, Tomohiro Suzuki^4^, Takeru Saito^1,5^, Shuntaro Hakozaki^1,6^, Manfred Wanner^7^, Ryota Koizumi^1^, Tatsuya Sakai^1^, Maribet Gamboa^8,9^, Toshihiko Tanaka ^1,10,11,12^, Akiko Ono^4,13^, Hoa Thanh Nguyen^14,15^, Yuta Saito^1,4^, Tetsuya Aoyama^11,12^, Katsuhiko Kojima^16^, Futoshi Suizu^17,18^, Kozo Watanabe^14^, Yoichiro Sogame^*†1^

*^1^Department of Applied Chemistry and Biochemistry, National Institute of Technology, Fukushima College, Iwaki, Fukushima, 970-8034, Japan*

*^2^Present address:* *Department of Chemistry and Biotechnology, Kochi University, Kochi, Kochi 780-8520, Japan*

*^3^Present address: College of Biological Sciences, University of Tsukuba, Tsukuba, Ibaraki 305-8577, Japan*

*^4^Center for Bioscience Research and Education, Utsunomiya University, Utsunomiya, Utsunomiya 321-8505, Japan*

*^5^Present address: College of Agri-Biological Resource Sciences, University of Tsukuba, Tsukuba, Ibaraki 305-8577, Japan*

*^6^Present address: Faculty of Symbiotic Systems Science, Fukushima University, Fukushima, Fukushima 960-1296, Japan*

*^7^Brandenburg University of Technology Cottbus-Senftenberg, Department of Ecology, D-03013 Cottbus,*

*Germany*

*^8^Department of Ecology, Faculty of Science, Universidad Catolica de la Santisima Concepcion, Alonso*

*de Ribera 2850, Concepcion, Chile*

*^9^ Centro de Investigación en Biodiversidad y Ambientes Sustentables (CIBAS), Universidad Católica de la Santísima Concepción, Alonso de Ribera 2850, Concepción, Chile*

*^10^Hamamatsu University School of Medicine, 1-20-1 Handayama, Higashi-ku, Hamamatsu, Shizuoka 431-3192, Japan*

*^11^Elements Chemistry Laboratory, RIKEN Cluster for Pioneering Research (CPR), Wako, Saitama 351-0198, Japan*

*^12^Ultrahigh Precision Optics Technology Team, RIKEN Center for Advanced Photonics (RAP), Wako, Saitama 351- 0198, Japan*

*^13^Present address: Faculty of Global Interdisciplinary Science and Innovation, Shizuoka University, Shizuoka, Shizuoka 422-8529, Japan*

*^14^ Center for Marine Environmental Studies (CMES), Ehime University, Matsuyama, Ehime 790-8577, Japan*

*^15^Present address: Department of Biomedical Sciences, School of Medicine and Health Sciences, University of North Dakota, Grand Forks, North Dakota, USA*

*^16^ Department of Microbiology and Immunology, Shinshu University School of Medicine, Matsumoto, Nagano 390-8621, Japan*

*^17^ Molecular Oncologic Pathology, Department of Pathology and Host-Defense, Faculty of 　Medicine, Kagawa University, Takamatsu, Kagawa 761-0793, Japan*

^18^ *Present address: Laboratory of Pathology, Department of Medical Technology, Kagawa Prefectural University of Health Sciences,Takamatsu, Kagawa 761-0123, Japan*

†: R. Saito and Y. Sogame contributed equally.**^*^**Corresponding; Yoichiro Sogame, National Institute of Technology Fukushima College, 30 Nagao Kamiarakawa Taira Iwaki, 970-8034 Japan. E-mail: sogame@fukushima-nct.ac.jp, gamegamesogamail@gmail.com, TEL: +81-246-46-0875.


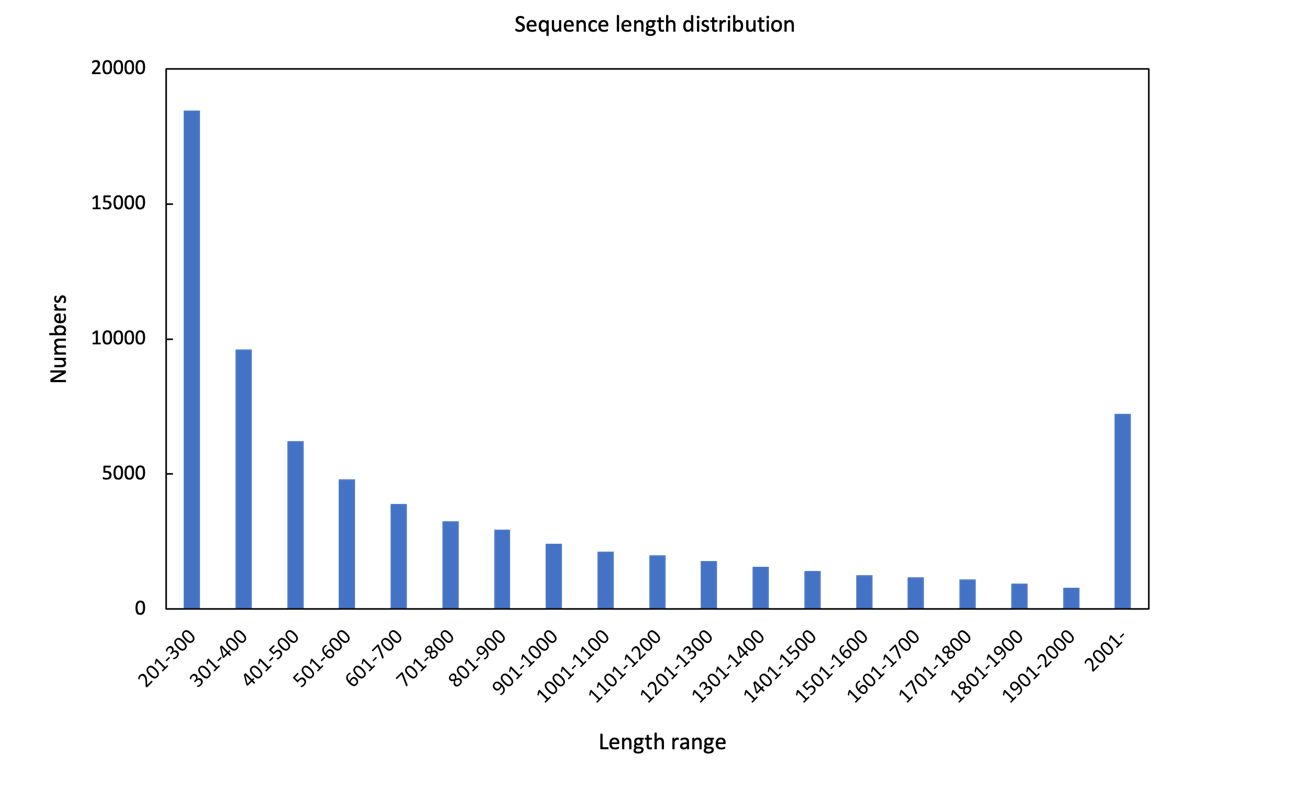


**Fig. S1.** Distribution of unigenes length from transcriptomic analysis of *C. cucullus* vegetative cell and cysts.

| **Gene id** | **Gene name** | **Primer F** | **Primer R** |
| --- | --- | --- | --- |
| Vegetative cell specific genes |  |  |  |
| TRINITY_DN26936_c0_g2_i1 | V-type proton ATPase 16 kDa proteolipid subunit | AATGTAGCGCGTTGTTTGCC | ATTTTCGCCGAAGCTCTTGG |
| TRINITY_DN24844_c0_g1_i1 | Ubiquitin-conjugating enzyme E2-16 kDa | AATTCCACTGGCAAGCAACC | TGGGGTGATAGATTCTGGTGAC |
| TRINITY_DN12541_c0_g1_i1 | Eukaryotic translation initiation factor 5A-3 | ATCTTTTCTTGGCCGACAGC | TGGCATTAAGGCTGCTTTCG |
| TRINITY_DN29395_c1_g1_i1 | Cell division control protein 48 homolog A | ATATGCCGCCAGACAAAGTG | TTGTTGTTGACCACCGCTTG |
| TRINITY_DN39536_c0_g1_i1 | Cathepsin D | AAAAGCCAGACTGCACCAAC | AACACCCAAAAGGCATTGGC |
| Cyst specific genes |  |  |  |
| TRINITY_DN29333_c1_g2_i1 | Calcium-dependent protein kinase 1 | TGGTGGTTTTTGCCTTCACG | ACAACCAGCCAACACAAGAC |
| TRINITY_DN34155_c0_g1_i1 | Growth arrest-specific protein 8 homolog | TTGGTCAATGAGCTCCAGAGAG | TTGGCACCAAAGGATCGAAC |
| TRINITY_DN33299_c0_g1_i1 | Parkin coregulated gene protein homolog | TTTGGGGGCTTGGTTTGTTC | AGCGAAACCGGAATCCAAAC |
| TRINITY_DN21381_c0_g1_i1 | ADP-ribosylation factor-like protein 3 | AGCGCCATCATCAAATCCTG | ACACAACTGATCACGGAAGC |
| TRINITY_DN480_c0_g1_i1 | Zinc finger protein 143 | ATGCAAACACGCAGGTTGTG | TTGGCACACAAAAGGTCTGG |
| Internal control genes |  |  |  |
| TRINITY_DN22777_c0_g1 | Transcription elongation factor SPT4 | TCCTTCGAAGCTTGGTGTTG | ATGCCTTACATGCCGTCTTG |
| GenBank, accession No. X94348.1 | Colpoda sp. gene encoding for alpha-tubulin, partial | CGGTAAGGAAGATGCTGCCA | GGGACGAGGTTGGTTTGGAA |
| CHS5 gene |  |  |  |
| TRINITY_DN26596_c0_g1 | RecName: Full=Cell fusion protein cfr1; AltName: Full=CHS5-related protein 1 | ACAAAACCGCAACTCCATCC | ACAAAACCGCAACTCCATCC |

Table S1. List of gene id, gene name, and primers.

|  |  |
| --- | --- |
| Sample | Clean reads |
| Vegetative | 13,518,200 |
| Cyst | 12,733,840 |

**Table S2.** Sequence results

| Total sequences | Total bases | Max sequence length (bp) | Min sequence length (bp) | Average length  (bp) | Median sequence length (bp) | N50 length  (bp) | (G+C)s | (A+T)s |
| --- | --- | --- | --- | --- | --- | --- | --- | --- |
| 73,011 | 65,639,138 | 17,589 | 201 | 899.03 | 544 | 1,443 | 39.59 | 60.41 |

**Table S3.** *De novo* sequence assembly

**Table S4.** All transcriptome data in this study. The file is provided separately in Excel format.

**Table S5.** Selected differentially expressed genes in vegetative cells and resting cysts. The file is provided separately in Excel format.

**Table S6.** Results of gene enrichment analysis. Yellow color enhanced GO. terms were significant differences between vegetative cells and cysts. The positive number log2 transformed fold change represent upregulation in vegetative cells, while the negative represents upregulation of cysts. The file is provided separately in Excel format.
